# Wedge-shaped microfluidic chip for circulating tumor cells isolation and its clinical significance in gastric cancer

**DOI:** 10.1186/s12967-018-1521-8

**Published:** 2018-05-23

**Authors:** Chaogang Yang, Nangang Zhang, Shuyi Wang, Dongdong Shi, Chunxiao Zhang, Kan Liu, Bin Xiong

**Affiliations:** 1 Department of Gastrointestinal Surgery & Department of Gastric and Colorectal Surgical Oncology, Zhongnan Hospital of Wuhan University, No.169 Donghu Road, Wuchang District, Wuhan, 430071 China; 2 Hubei Key Laboratory of Tumor Biological Behaviors, No.169 Donghu Road, Wuchang District, Wuhan, 430071 China; 3 Hubei Cancer Clinical Study Center, No.169 Donghu Road, Wuchang District, Wuhan, 430071 China; 4 College of Electronic and Electrical Engineering, Wuhan Textile University, No.1 Sunshine Avenue, Hongshan District, Wuhan, 430200 China; 5 School of Life Science and Technology, University of Electronic Science and Technology of China, No.4, Section 2, North Jianshe Road, Chengdu, 610054 China

**Keywords:** Circulating tumor cells, Cell capture, Wedge-shaped chip, Microfluidic, Gastric cancer

## Abstract

**Background:**

Circulating tumor cells (CTCs) have great potential in both basic research and clinical application for the managements of cancer. However, the complicated fabrication processes and expensive materials of the existing CTCs isolation devices, to a large extent, limit their clinical translation and CTCs’ clinical value. Therefore, it remains to be urgently needed to develop a new platform for achieving CTCs detection with low-cost, mass-producible but high performance.

**Methods:**

In the present study, we introduced a novel wedge-shaped microfluidic chip (named CTC-ΔChip) fabricated by two pieces of glass through wet etching and thermal bonding technique for CTCs isolation, which achieved CTCs enrichment by different size without cell surface expression markers and CTCs identification with three-color immunocytochemistry method (CK+/CD45−/Nucleus+). We validated the feasibility of CTC-ΔChip for detecting CTCs from different types of solid tumor. Furthermore, we applied the newly-developed platform to investigate the clinical significance of CTCs in gastric cancer (GC).

**Results:**

Based on “label-free” characteristic, the capture efficiency of CTC-ΔChip can be as high as 93.7 ± 3.2% in DMEM and 91.0 ± 3.0% in whole blood sample under optimized conditions. Clinically, CTC-ΔChip exhibited the feasibility of detecting CTCs from different types of solid tumor, and it identified 7.30 ± 7.29 CTCs from 2 mL peripheral blood with a positive rate of 75% (30/40) in GC patients. Interestingly, we found that GC CTCs count was significantly correlated with multiple systemic inflammation indexes, including the lymphocyte count, platelet count, the level of neutrophil to lymphocyte ratio and platelet to lymphocyte ratio. In addition, we also found that both the positivity rate and CTCs count were significantly associated with multiple clinicopathology parameters.

**Conclusions:**

Our novel CTC-ΔChip shows high performance for detecting CTCs from less volume of blood samples of cancer patients and important clinical significance in GC. Owing to the advantages of low-cost and mass-producible, CTC-ΔChip holds great potential of clinical application for cancer therapeutic guidance and prognostic monitoring in the future.

**Electronic supplementary material:**

The online version of this article (10.1186/s12967-018-1521-8) contains supplementary material, which is available to authorized users.

## Background

Circulating tumor cells (CTCs) are cancer cells that break away from the primary site and disseminate into blood stream. These “break away” cells, circulate and survive in the peripheral blood, potentially seed into distant organs and finally form the vital metastases—the main cause of cancer-related death. Recently, CTCs’ detection has been considered as a “liquid biopsy”, which could supply important information for clinical practice, such as treatment response monitor, potential metastasis prediction and prognosis evaluation in varieties of malignant tumors, including breast [1], prostate [2], gastric [3], colorectal [4] and lung cancer [5]. However, due to the extremely low concentration of CTCs (one in millions of blood cells) [6, 7], CTCs’ detection has always been technically challenging.

Over the past decades, numerous separation technologies had been developed based on different biological and physical properties of CTCs [8–17]. The CellSearch™ system (Veridex, LLC, Raritan, NJ, USA), depending on epithelial cell adhesion molecule (EpCAM) marker to enrich and isolate CTCs [18], was the first and only semi-automated CTCs enumeration assay approved by FDA for the diagnosis of metastatic breast, prostate, and colorectal cancers. Nevertheless, EpCAM-based methods may loss parts of CTCs undergoing epithelial-to-mesenchymal transition (EMT) [19], which might not appropriate to get a more comprehensive spectrum of CTCs. In addition, because of the expensive antigen, label-based methods were limited for clinical use. Recently, isolation by size of epithelial tumor cells (ISET) technology had been widely used as a new set of tools for CTCs capture and achieved higher CTCs detection sensitivity [10, 20, 21]. Based on this method, our ^os^ISET device [22] showed high efficiency of CTCs isolation in several types of solid tumor clinically. When ^os^ISET was clinically used, we found that the size heterogeneity of CTCs leads to miss parts of CTCs (diameter < 8 μm). For gastric cancer (GC), although several studies demonstrated that CTCs’ detection could be utilized for judging tumor stage, predicting patients’ survival and monitoring therapeutic response [23–25], but the drawbacks of the above methods limited its further clinical application to validate the significant role of CTCs.

Herein, we fabricated a low-cost wedge-shaped microfluidic chip (named CTC-ΔChip) to efficiently isolate CTCs from blood. Owning to the special structure of the microfluidic chamber, the size cut-off for enriching CTCs in the CTC-ΔChip was able to be designed as small as 5 μm [26] and CTCs even with slightly differential of biophysical properties could be entrapped and located at the microfluidic chamber with large space distribution. What’s more, the CTC-ΔChip still maintained a large loading volume of peripheral blood and quick sample processing. In this study, we demonstrated two aspects: (1) The feasibility and capture efficiency of CTC-ΔChip for CTCs detection from the whole blood samples of different types of solid cancer patients; (2) The potential clinical value and significance of CTCs isolated by it in GC patients.

## Methods

### Preparation of CTC-ΔChip

**Fig. 1:**
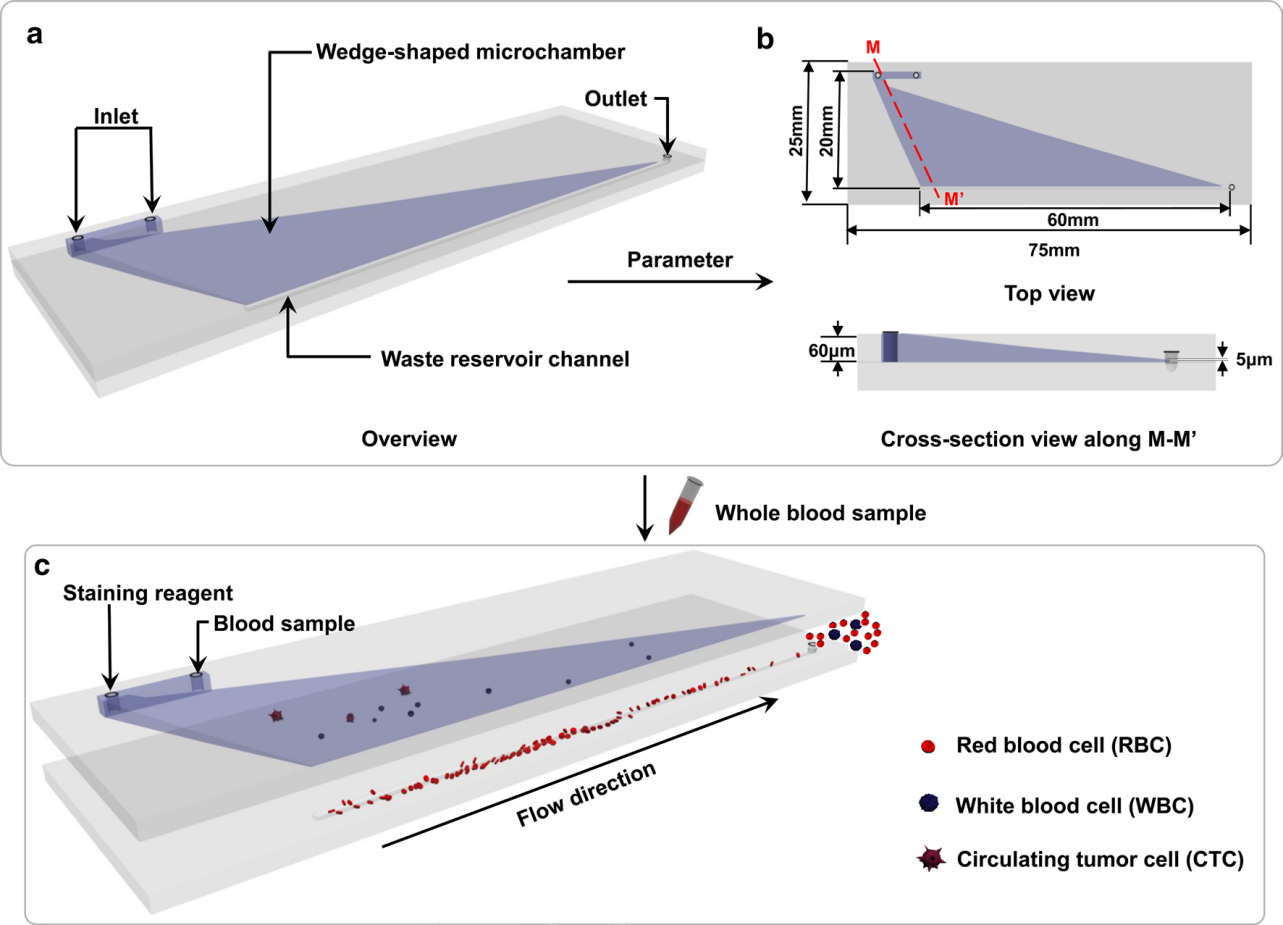
Schematic diagram of CTCs isolation using the wedge-shaped microfluidic chip. **a** Overview of the wedge-shaped microfluidic chip; **b** detailed structural parameters of the microfluidic chip; **c** schematic diagram of CTCs isolation using the microfluidic chip with a wedge-shaped microchamber

CTC-ΔChip was fabricated by wet etching technique and thermal bonding technique. Details were showed in Additional file 1. As shown in Fig. 1a, CTC-ΔChip was composed of a wedge-shaped microchamber, two inlets, a linear reservoir and one outlet. The wedge-shaped microchamber was the core functional part of the CTC-ΔChip to isolate CTCs from patient’s whole blood. The detailed structure parameters of CTC-ΔChip were shown in Fig. 1b, and the schematic diagram of CTC isolation using the CTC-ΔChip was shown in Fig. 1c. To investigate how the outlet height (size cut-off for enrich CTCs) of microchamber affected the capture efficiency, CTC-ΔChip with different outlet heights (4, 5, 6, 7, 8 μm, respectively) were fabricated. Finally, CTC-ΔChip was assembled into a small microfluidic device, the detailed structure of which was shown in Additional file 2.

### Cancer cell and human blood sample preparation

BGC823 (human gastric cancer cell), HCT116 (human colorectal cancer cell), PC3 (human prostate cancer cell) and SKBR3 (human breast cancer cell) were obtained from Hubei Key Laboratory of Tumor Biological Behaviors. Cells were cultured in Dulbecco’s modified eagle medium (DMEM, Hyclone, Thermo scientific, USA) added with 10% fetal bovine serum (Sigma, USA) and 1% penicillin/streptomycin at 37 °C in 5% humidified CO_2_ incubator. Adherent cells were released with 0.25% (w/v) trypsin (Gibco, USA), resuspended in DMEM medium, assessed for concentration with a hemocytometer, and rocked gently on a shaker 5 min prior to experiments. Whole blood samples from healthy donors and patients were obtained from Department of Clinical Laboratory, Zhongnan Hospital of Wuhan University according to a protocol by the Institutional Review Board (IRB). All blood specimens were collected into the vacutainer tubes (BD, New Jersey, USA) containing EDTA-K2 and finished the experiments within 3 h. All the participants have provided their written informed consent to participate in this study. This research was approved by the Medical Ethical Committee of Zhongnan Hospital.

### Optimization assays

Spiked samples containing target cells (BGC823, ~ 100 cells 2 mL^−1^ DMEM, pre-stained with CK) were injected into CTC-ΔChip with different outlet heights (4, 5, 6, 7, 8 μm, respectively) at flow rate of 200 μL/min to investigate the effect of outlet height on the capture efficiency. Finally, the spiked samples were injected into CTC-ΔChip with 5 μm outlet height at different flow rate (50, 100, 200, 300, 400, 600, 800 μL/min, respectively) to investigate the effect of flow rate on the capture efficiency.

### Sample processing and performance validation

BGC823, HCT116, PC3 and SKBR3 cells were spiked into DMEM medium and blood sample at concentrations of 200 cells per 2 mL. Just prior to cell capture, 4% paraformaldehyde (PFA) solution was added into whole blood samples in a 1:10 v/v ratio, and incubated for 10 min at room temperature. Then, the spiked samples were introduced into the CTC-ΔChip by use of syringe pump in optimal cell-capture condition. Each test had been repeated for three times. After sample loading, CTC-ΔChip was rinsed with PBS solution at 200 μL/min for 10 min. After rinsing, followed by staining of three-color immunocytochemistry method for CK, CD45 and nucleus, specifically captured cancer cells were identified and counted on the substrates. Finally, we imaged and enumerated targeted cells using a high-content automatic screening system (CX7, CellInsight, ThermoFisher Scientific, USA).

### Cell capture from healthy and patient’s blood samples

Cell capture efficiency was validated using the CTC-ΔChip to capture target cells (BGC823) at concentrations of 50, 100, 150, 200 cells per 2 mL from the two kinds of sample: (1) 2 mL DMEM, and (2) 2 mL human blood (healthy donor). Patient blood samples (2 mL) were processed by the CTC-ΔChip according to the procedure described above. Captured cells were rinsed with PBS and then stained by three-color immunocytochemistry method for CK, CD45 and nuclear staining.

### Data collection

Clinicopathologic data of patients were collected from hospital information system, including age, gender, tumor location, pathological type, differentiation degree, lymphovascular invasion, perineural invasion, tumor depth, lymph node status, metastasis status, TNM stage, and the level of Ki-67 and tumor markers. The clinicopathological classifications of cancers were determined according to the 7th edition of the American Joint Committee on Cancer/International Union Against Cancer tumor-node-metastasis (TNM) classification system [27].

### Statistical analysis

Categorical data displayed in a contingency table were analyzed using Fisher’s exact test. Continuous data were analyzed by Mann–Whitney test. Nonparametric correlation analysis used the method of Spearman rank correlation analysis. All statistical analyses were performed with the IBM SPSS 22.0 statistical software package (IBM Inc.). P < 0.05 was considered statistically significant.

## Results

### Characterization and optimization of CTC-ΔChip

**Fig. 2:**
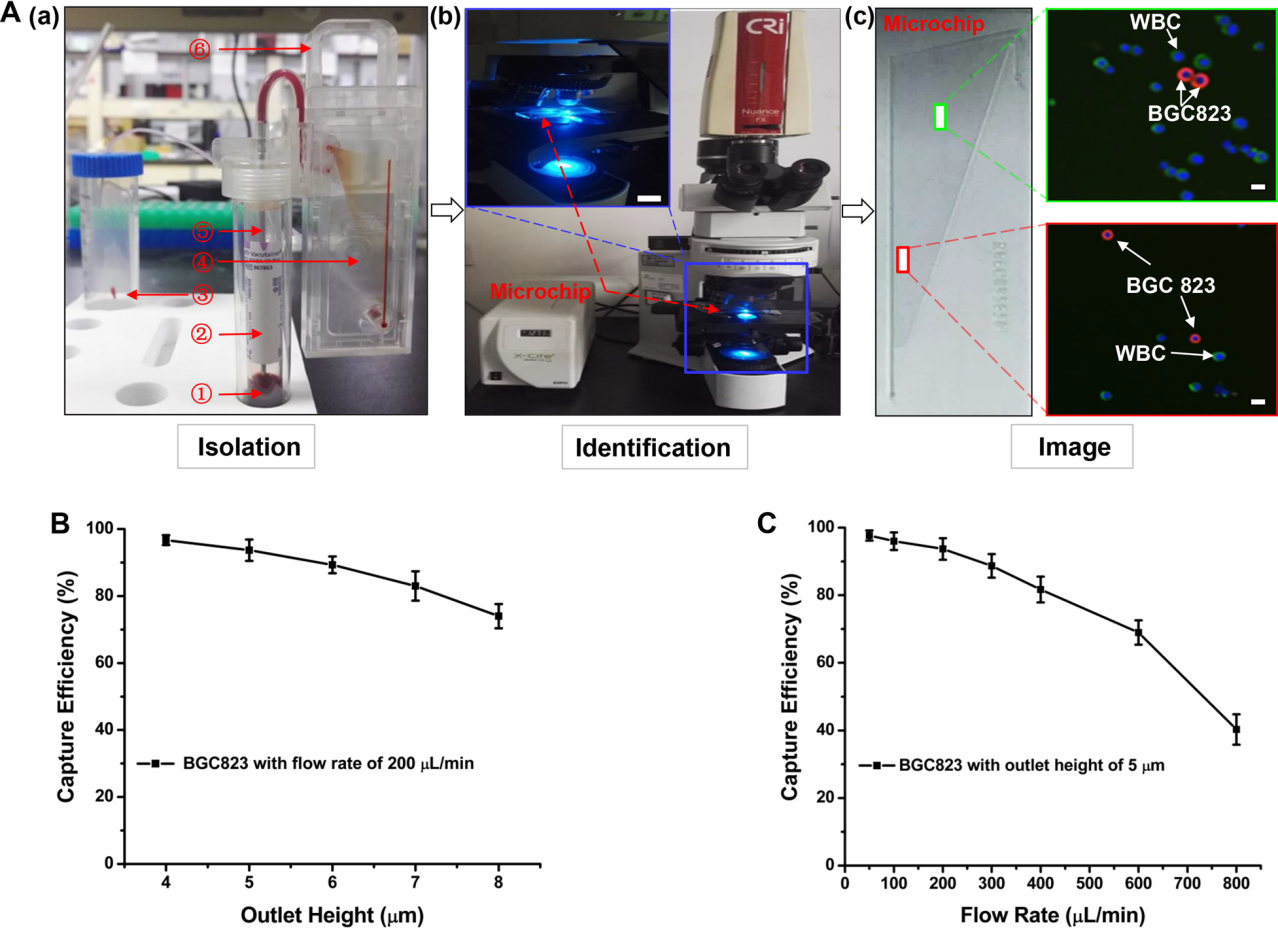
Optimization assays of the microfluidic chip. **A** Flow chart of the optimization assays: (a) isolating BGC823 cancer cells from blood sample using the microfluidic chip: ➀ blood sample, ➁ sampling tube, ➂ waste tube, ➃ microfluidic chip, ➄ sampling needle, ➅ clamping device; (b) Identification cancer cells captured by the microfluidic chip with three-color immunofluorescence staining (Pan-CK, red; FITC-CD45, green; DAPI nuclear staining, blue) using fluoresce microscope (scale bar, 10×); and (c) Three-color immunofluorescence image of BGC823 cancer cell (CK+, CD45− and DAPI+) and WBC (CK−, CD45+ and DAPI+) in two different height areas (red area: about 1 μm; green area: about 4 μm) of the chip. Scale bar, 100 μm; **B** the capture efficiency of the microfluidic chip at different outlet heights (4, 5, 6, 7, 8 μm, respectively); **C** the capture efficiency of the microfluidic chip at different flow rates (50, 100, 200, 300, 400, 600, 800 μL/min). The error bar represents standard deviation from three repeats

Figure 1 illustrated the schematic workflow of CTC-ΔChip to efficiently enrich the CTCs from whole blood samples based on the biophysical property. The wedge-shaped microchamber in CTC-ΔChip was the core function part. The height of the microchamber in CTC-ΔChip was designed to gradually decrease (Fig. 1b). The integrated process of CTCs isolation using the CTC-ΔChip was shown in Additional file 3. To obtain high capturing efficiency of CTCs and ensure the minimum loading volume of blood sample (2 mL) at the same time, optimization studies were conducted to determine a reasonable outlet height (Fig. 2A). Blood samples were collected and stored in 4 °C for 6, 24 and 48 h individually. The loading volume of patient blood sample was measured. When the cut-off size of CTC-ΔChip was 5 μm, the minimum loading volume of blood sample was more than 10, 7.6 and 3.6 mL (n = 5). Although the capture efficiency of BGC823 cells in DMEM decreased from around 96.7–74% with outlet height increasing from 4 to 8 μm, it’s still more than 93.7% in outlet height of 5 μm (Fig. 2B). Considering of capture efficiency and minimum blood loading volume, the optimal cut-off size of the CTC-ΔChip would be 5 μm. The relationship of capture efficiency and flow rate had been studied. As we known, the flow rate would decide the whole completion time of sample processing. Thus, we validated the cell capture efficiency of CTC-ΔChip using optimized outlet height in different flow rates. As observed in Fig. 2C, the capture efficiencies decreased from 97.7 to 40.3% when the flow rates increased from 50 to 800 μL/min. When the flow rate was 200 μL/min, the capture efficiency was up to 93%. In order to achieve the balance of completion time and capture efficiency, 200 μL/min was chosen for the optimal flow rate of the CTC-ΔChip.

### Performance evaluation of CTC-ΔChip

**Fig. 3:**
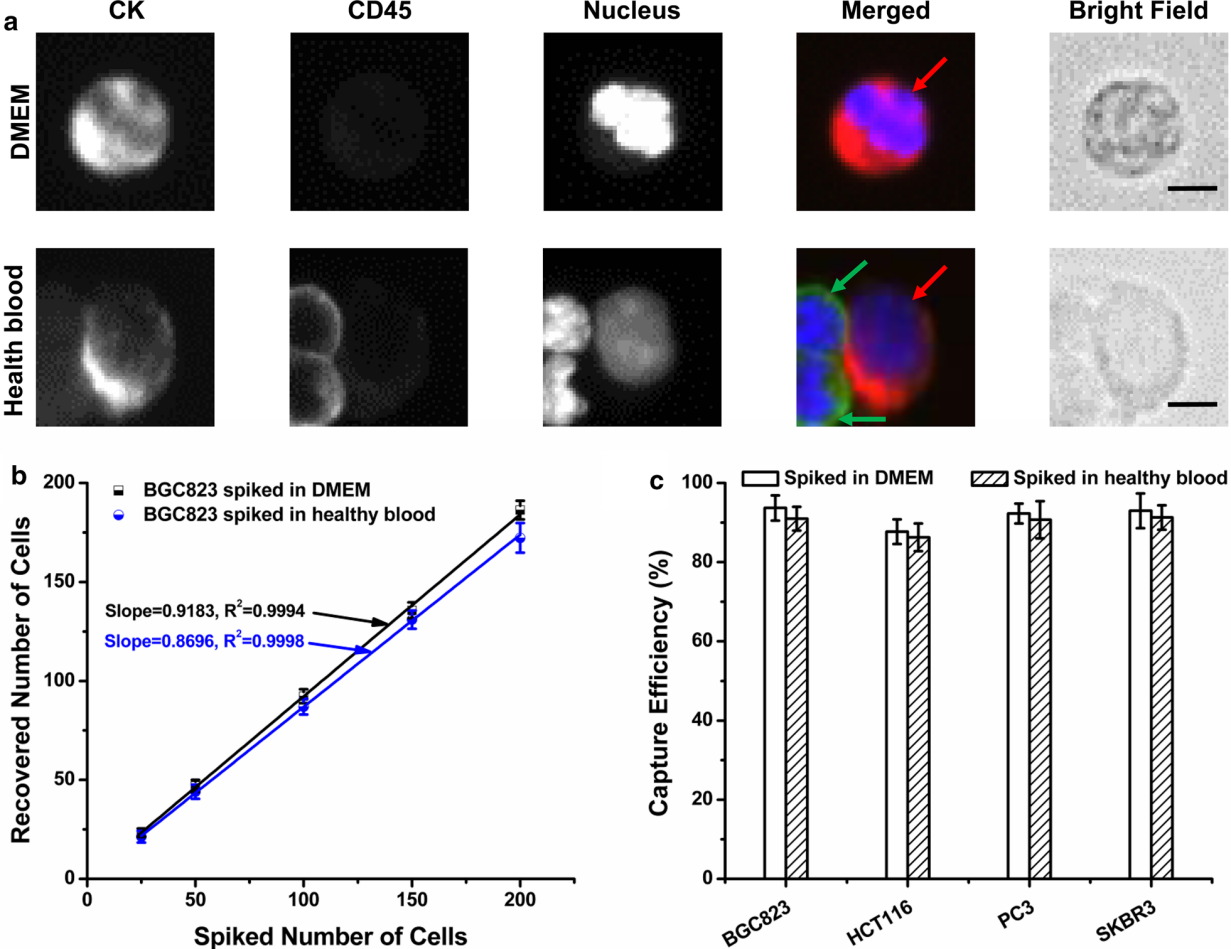
Capturing performance of the CTCs enrichment platform equipped with a wedge-shaped microchamber. **a** The fluorescent micrographs of BGC823 captured from DMEM and healthy blood sample. Three-color immunocytochemistry method based on CK, CD45, and nuclear staining was applied to identify and enumerate BGC823 (red arrow) from WBCs (green arrow). Scale bars are 10 μm; **b** the recovered number of BGC823 was validated from DMEM and healthy blood sample with different spiking level (25, 50, 100, 150, 200 cells/2 mL, respectively); **c** the capture efficiency of four tumor cells lines (BGC823, HCT116, PC3, SKBR3) spiked in DMEM and healthy blood samples. All experiments were performed under optimal condition. The error bar represents standard deviation from three repeats

Under optimized condition, series of studies were conducted to assess the capture performance of CTC-ΔChip. As shown in Fig. 3b, the average capture efficiencies of BGC823 cells were around 93% from DMEM and around 87% from healthy blood samples, respectively. Meanwhile, the recovered number showed excellent linear relationship with spiked number of cells in DMEM (R^2^ = 0.9994) and healthy blood samples (R^2^ = 0.9998). Moreover, the capture efficiencies of detecting spiked BGC823, HCT116, PC3 and SKBR3 cells into DMEM were (93.7 ± 3.2), (87.7 ± 3.1), (92.3 ± 2.5) and (93.0 ± 4.4)%, while the capture efficiencies of spiked BGC823, HCT116, PC3 and SKBR3 cells into blood sample were (91.0 ± 3.0), (86.3 ± 3.5), (90.7 ± 4.7) and (91.3 ± 3.1)% (Fig. 3c). The fluoresce images of BGC823 were shown in Fig. 3a. Based on the “label-free” technology, CTC-ΔChip exhibited high efficiency in isolating tumor cells in different type of cancer cell lines.

### Feasibility of detecting clinical samples using CTC-ΔChip

To validate the feasibility of detecting CTCs from clinical samples using our CTC-ΔChip, we collected 2 mL peripheral blood samples from 76 cancer patients, including 20 breast cancers, 15 lung cancers, 13 esophageal cancers, 16 gastric cancers and 12 colorectal cancers. CTCs were isolated, stained and enumerated with CTC-ΔChip according to the steps mentioned above. The detailed clinical information of them were recorded in Additional file 4, the cellular morphology and cell nucleus were shown clearly by three-color immunocytochemistry method (Additional file 5), and the enumeration results showed CTCs were detected from 53 patients (69.7%) with the number of CTCs ranged from 0 to 30 (Additional file 6), while none of CTCs was detected in 25 healthy donors. Owing to base on the physical properties of CTCs, CTC-ΔChip showed high performance for capturing CTCs from clinical samples.

### Clinical Significance of CTCs Detected by CTC-ΔChip in GC

To study its clinical significance of CTCs detected by our CTC-ΔChip in GC, 2 mL blood samples from 40 patients (Gender: 25 males and 15 females; Mean age: 62.1 [45–74] years) were collected before any treatment (Table 1). All patients included in the study were firstly diagnosed via pathological examination by endoscopic biopsy and hospitalized in Zhongnan Hospital of Wuhan University between June 1st 2017 and October 1st 2017, without a history of venous thrombosis or anticoagulation therapy, cardiovascular and cerebrovascular disease, acute or chronic inflammatory disease, blood system or immune system defects, previous malignancy.

**Table 1:** Gastric cancer patient characteristics (n = 40)

Group	Patients (n)	Percentage (%)	Count of CTCs+ patients (n)	Count of CTCs (mean ± SD)	CTCs positive rate (%)
Sex					
Male	25	62.5	19	7.38 ± 7.58	76.0
Female	15	37.5	11	7.21 ± 7.17	73.3
Age (years)					
≥ 60	22	55.0	17	6.65 ± 6.66	77.27
< 60	18	45.0	13	8.18 ± 8.20	72.22
Tumor location					
Cardia	12	30.0	9	7.50 ± 7.65	75.0
Body	14	35.0	10	5.71 ± 5.44	71.4
Pylorus	14	35.0	11	8.71 ± 8.69	78.6
Histologic subtype					
Adenocarcinoma	17	42.5	13	5.71 ± 5.16	76.5
Mucinous adenocarcinoma	11	27.5	8	7.73 ± 6.72	72.7
Signet-ring cell carcinoma	12	30.0	9	9.17 ± 10.04	75.0
Differentiation					
Well	12	25.0	7	3.17 ± 3.61	58.3
Moderate	12	22.5	9	6.42 ± 5.55	75.0
Poor	16	52.5	14	11.06 ± 8.76	87.5
Lymphovascular invasion					
Without	18	45.0	10	2.17 ± 2.38	55.6
With	22	55.0	20	11.50 ± 7.29	90.9
Perineural invasion					
Without	19	47.5	11	2.84 ± 3.44	57.9
With	21	52.5	19	11.33 ± 7.56	90.5
TNM stage^a^					
Stage I	5	12.5	2	0.80 ± 1.30	40.0
Stage II	12	30.0	7	2.67 ± 2.64	58.3
Stage III	16	40.0	14	7.88 ± 4.33	87.5
Stage IV	7	17.5	7	18.57 ± 7.59	100.0
Ki-67					
≥ 50%	25	62.5	22	10.60 ± 7.34	88.0
< 50%	15	37.5	8	1.80 ± 2.01	53.3
Total	40	100.0	30	7.30 ± 7.29	75.0

*CTCs* circulating tumor cells

^a^7th edition of AJCC/UICC classification system

**Fig. 4:**
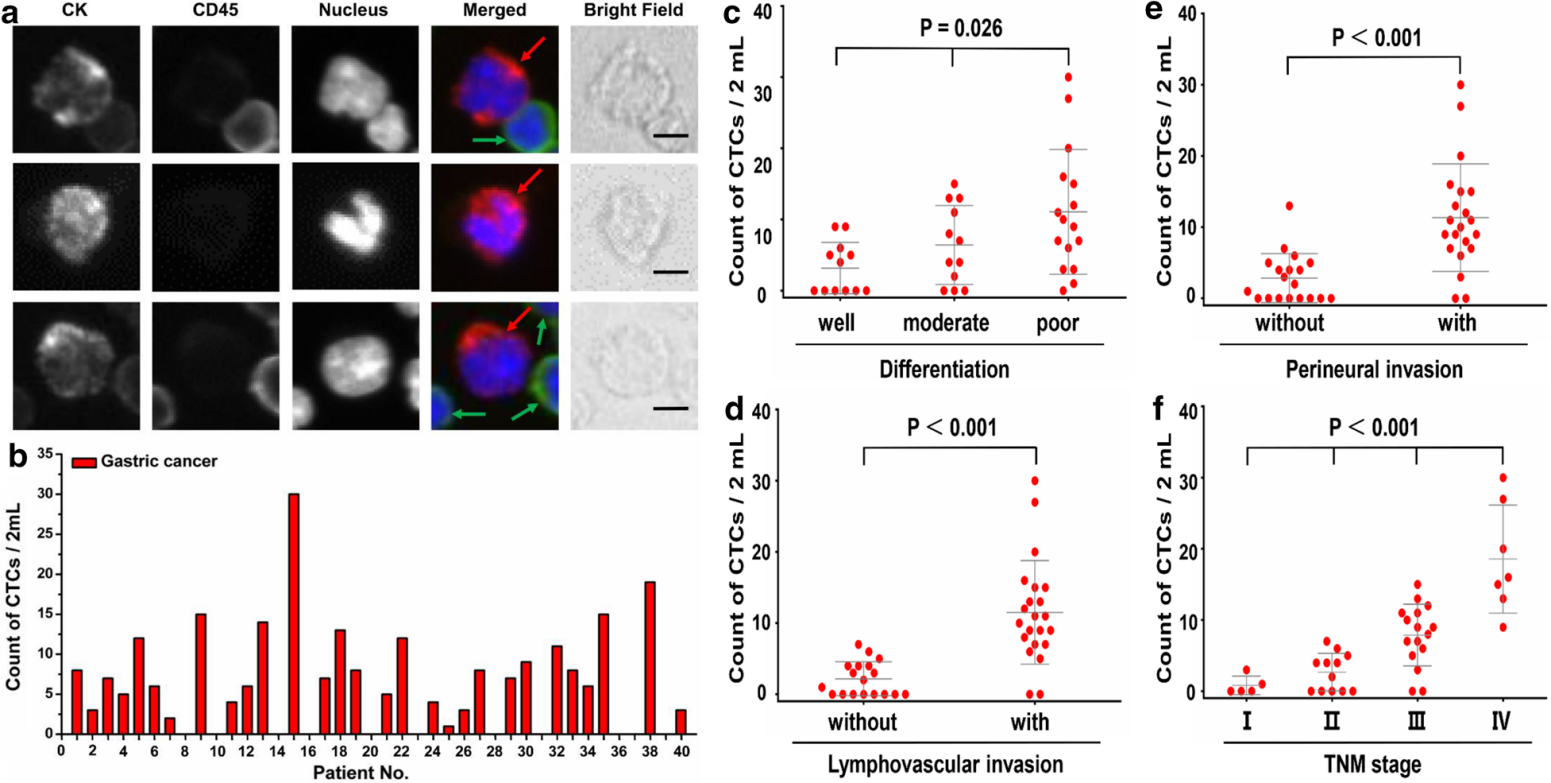
Result of CTCs isolation from 40 GC patients. **a** CTCs detected from a GC patient. Three-color immunocytochemistry method based on CK, CD45, and nuclear staining was applied to identify and enumerate CTCs (red arrow) from non-specifically trapped WBCs (green arrow). Scale bars are 10 μm; **b** CTCs enumeration results obtained from 40 GC patients. Scatter plot for CTCs number of GC patients; **c** different tumor differential status; **d** with and without lymphovascular invasion; **e** with and without perineural invasion; **f** different TNM stage, each red dot stands for one GC patient. The error bars represent standard error of the mean

After CTCs capture and staining in the CTC-ΔChip, the combined information was utilized to delineate CTCs cells (CK+, CD45−, Hoechst+) from WBCs (CK−, CD45+, Hoechst+) and cellular debris (Fig. 4a). Then we summarized the counts of CTCs in Fig. 4b and CTCs-positive rate in Table 2. The counts of isolated CTCs ranged from 0 to 30 per 2 mL, with an average of 7.30 ± 7.29 (Mean ± SD). Statistical analyses were used to illustrate the relationship between CTCs and clinical characteristics of GC patients. The results showed that both CTCs counts and CTCs’ positive rate detected were significantly different in patients with different status of tumor differentiation (P = 0.026; P = 0.030, respectively) (Fig. 4c), lymphovascular invasion (P < 0.001; P = 0.025, respectively) (Fig. 4d), perineural invasion (P < 0.001; P = 0.028, respectively) (Fig. 4e), TNM stage (P < 0.001; P = 0.033, respectively) (Fig. 4f) and Ki-67 level (P < 0.001; P = 0.024, respectively) (Table 2). Moreover, Spearman rank correlation analysis showed that CTCs counts were highly correlated with tumor differentiation (r = 0.432, P = 0.005), lymphovascular invasion status (r = 0.643, P < 0.001), perineural invasion status (r = 0.725, P < 0.001), Ki-67 level (r = 0.677, P < 0.001) and TNM stage (r = 0.789, P < 0.001) (Table 2). However, neither the positivity rate of CTCs nor the count of CTCs was significantly correlated with age, sex, tumor location, histologic subtype (Table 2). For tumor markers, there was significant difference for the level of carcinoembryonic antigen (CEA) between CTCs’ positive and CTCs’ negative group (p = 0.002), nevertheless, the significant difference did not exist in the other tumor markers level, such as carbohydrate antigen-153 (CA153), carbohydrate antigen-199 (CA199) and carbohydrate antigen-724 (CA724) (P > 0.05, respectively) (Additional file 7). As the result illustrated, CTC-ΔChip had shown high-performance in capturing CTCs from clinical samples. CTCs could be detected in 30 GC patients (75%) from 40 GC patients at various stages with an average of 7.30 ± 7.29. Even in patients of early stage (stage I and II, 52.9%) and well differentiated (30%), which exhibited a high capturing efficiency. Furthermore, CTC-ΔChip also showed the great potential for clinical application in detecting CTCs. Both the positivity rate and the counts of CTCs were positively correlated with lymph node metastasis status and TNM stage, which implied that CTCs could be used as the supplementary to evaluate the degree of disease progression and predict the prognosis of patients.

**Table 2:** The relationship of CTCs and clinicopathological variables of gastric cancer patients

Variable	CTCs positive rate	Count of CTCs	
	P^a^	P^b^	P^c^
Sex	1.000	0.913	0.915, r = 0.017
Age	0.731	0.553	0.560, r = 0.095
Tumor location	0.823	0.702	0.684, r = 0.684
Histologic subtype	0.975	0.748	0.511, r = 0.107
Differentiation	0.030	0.026	0.005, r = 0.432
Lymphovascular invasion	0.025	< 0.001	< 0.001, r = 0.643
Perineural invasion	0.028	< 0.001	< 0.001, r = 0.725
TNM stage^d^	0.033	< 0.001	< 0.001, r = 0.789
Ki-67	0.024	< 0.001	< 0.001, r = 0.677

*CTCs* circulating tumor cells

^a^P value from Fisher’s exact test

^b^P value from the Mann–Whitney *U* test

^c^P value from Spearman rank correlation analysis

^d^7th edition of AJCC/UICC classification system

**Fig. 5:**
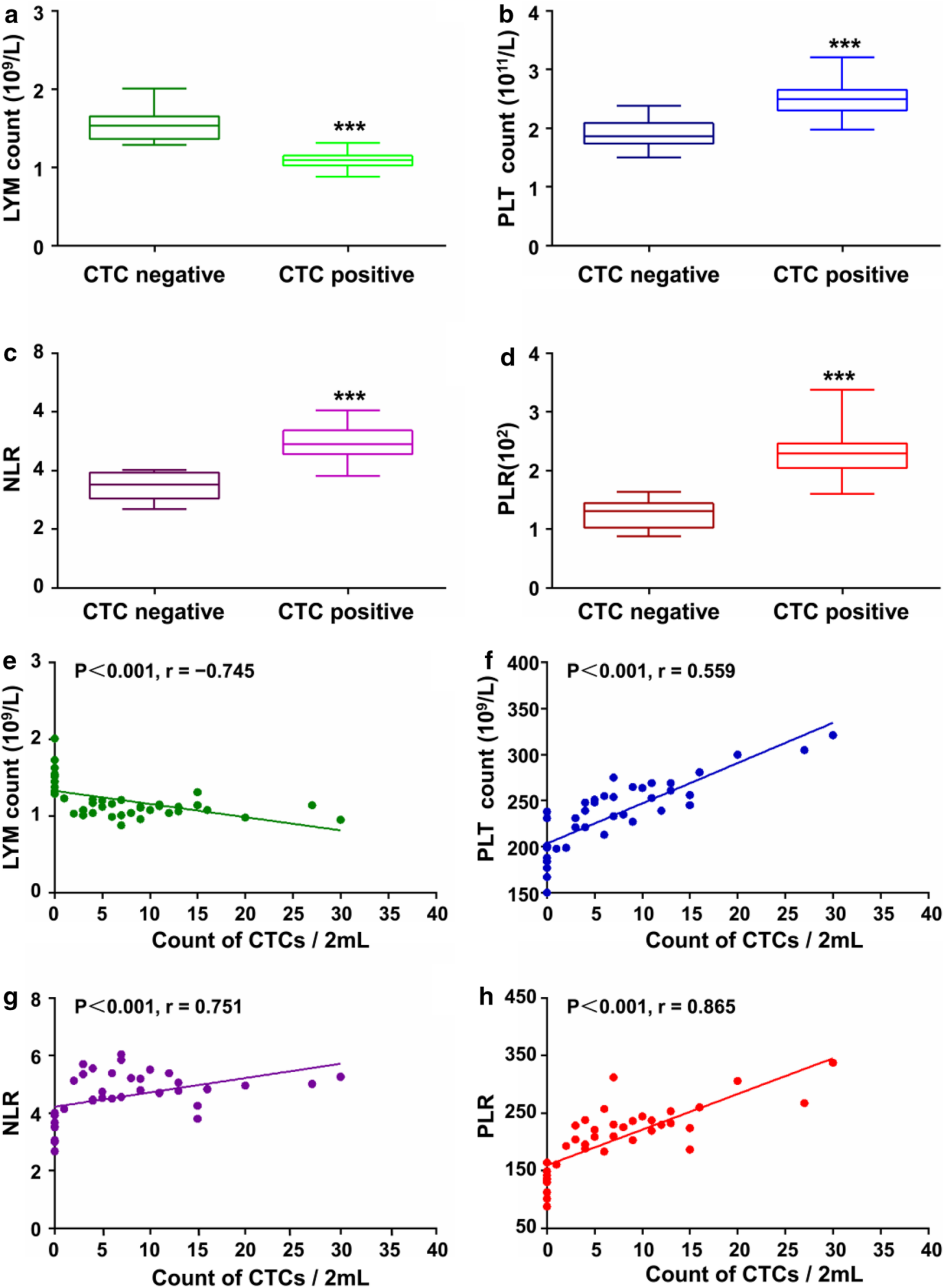
Association of CTCs and blood microenvironment indexes. The difference of **a** LYM count, **b** PLT count, **c** NLR and **d** PLR between CTCs-positive and negative group. ***Represents P < 0.001. The relationship of CTC count and **e** LYM count, **f** PLT count, **g** NLR and **h** PLR

Furthermore, we investigated the relationship between CTCs count and blood systemic inflammation indexes (Table 3). Interestingly, we found that the lymphocyte count, platelet (PLT) count, the level of neutrophil to lymphocyte ratio (NLR) and platelet to lymphocyte ratio (PLR) were statistically significant between CTCs-positive and CTCs-negative group (P < 0.001, respectively; Fig. 5a–d), further Spearman rank correlation analysis showed that there was a positive correlation between the CTCs count and PLT count, NLR and PLR (r = 0.559, P < 0.001; r = 0.751, P < 0.001; r = 0.865, P < 0.001, respectively; Fig. 5e–h). However, there was no significant difference between CTCs positive and negative group for leukocyte, neutrophil or monocyte (P = 0.766; P = 0.691; P = 0.587, respectively; Table 3).

**Table 3:** Association between CTCs and blood microenvironmental indexes

Parameter	CTCs+ (n = 30)	CTCs− (n = 10)	P value
Immune cell			
Leukocyte count (10^9^/L)	7.28 ± 0.06^a^	7.26 ± 0.06	0.766^b^
Neutrophil count (10^9^/L)	5.24 ± 0.03	5.24 ± 0.02	0.691^b^
Lymphocyte count (10^9^/L)	1.01 ± 0.02	1.57 ± 0.02	< 0.001^c^, r = − 0.745
Monocyte count (10^9^/L)	0.40 ± 0.01	0.39 ± 0.01	0.587^b^
Systemic inflammatory factor			
PLT (10^9^/L)	237.92 ± 8.12	190.14 ± 6.17	< 0.001^c^, r = 0.559
NLR	5.20 ± 0.08	3.36 ± 0.05	< 0.001^c^, r = 0.751
PLR	236.69 ± 9.26	125.97 ± 3.94	< 0.001^c^, r = 0.865

*CTCs* circulating tumor cells

^a^Mean ± SEM

^b^P values from the Mann–Whitney test

^c^P values from Spearman rank correlation analysis

## Discussion

In our study, we developed a novel wedge-shaped microfluidic chip (CTC-ΔChip) for CTCs isolation, which achieved to high efficiently isolate CTCs from less volume of patients’ blood without clogging issue. In the structural of CTC-ΔChip, the pillar-array fluid distributor made the velocity distribution of blood so linear in the entire wedge-shaped microchannel that the microchannel is not clogged by blood cells and the design of wedge-shaped microchannel provided a wide outlet also could solve the problem of outlet clogging. On this basis, benefiting to the biophysical properties of CTCs, such as stiffer, larger sizes, larger nuclear/cytoplasmic ratios, deformability and non-spherical bioparticles characteristic compared with other blood cells, red blood cell (RBC) would flow and randomly rotate through the microchamber, when the height of the microchamber was larger than the size of RBC. When the height of the microchamber was smaller than the diameter of RBC, RBC would be laid down due to their disk-like shape in the process of sample processing. And, due to the cut-off size of the microchamber was larger than the thickness of RBC, RBC would smoothly go through the microchamber without clogging the microfluidic chip [28, 29]. Additionally, almost 99.9% WBCs would go through CTC-ΔChip instead of CTCs, even the cut-off size of the CTC-ΔChip was smaller than the diameter of white blood cell (WBC). What’s more, the rest subgroup of WBC left in the microchamber would be separated from CTCs with huge space resolution due to utilizing size-space amplification by the wedge-shaped microchamber. In theory, the microchamber would amplify 1 μm size differential to 1 mm distance differential.

Importantly, CTC-ΔChip was just fabricated by two common glass slides through wet etching technique and thermal bonding technique. Owing to the cheap raw materials and simple production processes, our CTC-ΔChip showed the advantages of low-cost and mass-producible, and had more potential for clinical translation. Moreover, compared with the previous microfluidic devices, our chip had incorporated with automated high-resolution fluorescence imaging into workflow, which achieved of detecting, identifying and enumeration for CTCs efficiently and automatically, shortened the time of processing samples and reduces the error of manual operation.

Additionally, in this study, our CTC-ΔChip also show preliminary clinical value in GC. The degree of tumor differentiation, as one of prognostic indicator for GC patients, was positively correlated with both the positivity rate and the counts of CTCs, this correlation was in correspondence with the fact that poor differentiation of tumor had the greater risk of metastasis. The correlation of lymphovascular invasion and CTCs was another part of the results needed to be discussed. Lymphovascular invasion had been proved to be an important prognostic factor of GC [30, 31]. However, in our study, data showed that both the positivity rate and CTCs count in patients with lymphovascular invasion were higher than those in patients without lymphovascular invasion, this correlation implied that lymphovascular invasion may also be a source of systemic spread of CTCs, and it might contribute to distant metastasis. Perineural invasion, as a risk factor and another prognostic factor of GC patients [32, 33], was positively correlated with both the positivity rate and the count of CTCs in our study, which implied that CTCs might be served as another high-risk factor of GC to guide clinical practice.

Noteworthy, to our knowledge, our study firstly demonstrated the relationship of CTCs and blood microenvironment indexes of GC patients under the precondition of strictly setting the inclusion criteria. Theoretically, CTCs fall off from the primary lesion into the blood circulation every day [34], most of which were eliminated in the process of interacting with the blood microenvironment and only a fairly few survived in PB. Immune cells including lymphocytes, monocytes/macrophages and neutrophils, as one of component of the blood microenvironment, played an important role during this process as the previous studies reported [35, 36]. Therefore, combining lymphocytes and CTCs might be an indicator to estimate immune condition, guide immunotherapy and predict prognosis for cancer patients. In recent years, clear evidences showed that the systemic inflammation reaction and the interaction between various inflammatory cells and the extra-cellular matrix played a crucial role in the tumor microenvironment of tumorigenesis, progression and metastasis [37–39]. Laboratory parameters that could reflect the status of systemic inflammation, had been investigated as systemic inflammatory markers, such as PLR, NLR and PLT counts [40, 41]. Previous evidences suggested that PLT might participate in the inflammatory reaction by facilitating neutrophils adhesion to endothelium through releasing chemokines and cytokines [42] and promoting tumor progression through facilitation of neoangiogenesis, production of adhesion molecules and increase of early metastatic niches [43]. The lymphocytes response was a major factor in the suppression of cancer progression [44]. The mechanisms underlying neutrophil response in tumor growth and metastasis included releasing of reactive oxygen species or nitric oxide and remodeling of the extracellular matrix [45]. Moreover, a series of meta-analysis had proved that all of them were associated with the prognosis of GC [46–48]. Based on these results, we speculated that systemic inflammatory reaction might affect the progression and metastasis of GC by the activities of CTCs, however, the specific mechanisms needed further study to clarify.

## Conclusions

In summary, we had successfully developed a low-cost CTC-ΔChip for isolation and identification of CTCs with high-performance. Our results did not only suggest that CTC-ΔChip had sufficient specificity and sensitivity, but also exhibited a robust platform that could accept patient’s blood store for 48 h in 4 °C without clogging issues. More importantly, our CTC-ΔChip had showed important clinical value in GC patients. Taken together, through CTCs’ enumeration, CTC-ΔChip hold great potential of clinically translating to monitoring cancer prognosis and guiding individualized treatment in the future.

## Additional files

**Additional file 1.** The detail process of preparation of CTC-ΔChip.

**Additional file 2: Figure S1.** Detailed structure of the microfluidic device.

**Additional file 3.** Integrated process of CTCs isolation using the CTC-ΔChip.

**Additional file 4: Table S1.** Detailed clinical information and detected CTCs counts of 76 cancer patients.

**Additional file 5: Figure S2.** CTCs images of various type of cancers.

**Additional file 6: Figure S3.** Number of captured CTCs from blood samples of various type of cancer patients.

**Additional file 7: Table S2.** Association between CTCs and tumor markers.

## Abbreviations

CTCs: circulating tumor cells
GC: gastric cancer
PLT: platelet
NLR: neutrophil to lymphocyte ratio
PLR: platelet to lymphocyte ratio
EpCAM: epithelial cell adhesion molecule
EMT: epithelial-to-mesenchymal transition
ISET: isolation by size of epithelial tumor cells
DMEM: Dulbecco’s modified eagle medium
IRB: Institutional Review Board
PFA: paraformaldehyde
TNM: tumor-node-metastasis
CEA: carcinoembryonic antigen
CA153: carbohydrate antigen-153
CA199: carbohydrate antigen-199
CA724: carbohydrate antigen-724
RBC: red blood cell
WBC: white blood cell
CK: cytokeratin
