# Supplementary material for: Wedge-shaped microfluidic chip for circulating tumor cells isolation and its clinical significance in gastric cancer
Source: J Transl Med. 2018 May 23;16:139. doi: 10.1186/s12967-018-1521-8 (PMC5966930; doi:10.1186/s12967-018-1521-8)
Supplement: Supplementary file 1 — Additional file 1. The detail process of preparation of CTC-ΔChip. [file 12967_2018_1521_MOESM1_ESM.docx]

**Additionall file 1**

**The detail process of preparation of CTC-ΔChip**

The CTC-ΔChip was fabricated by wet etching technique and thermal bonding technique. First, a chemically corrosion-resistant adhesive tape was coated on standard glass slide (75mm×25mm), then the laser ablation system was utilized to transfer the microchannel design onto the adhesive tape. Second, the glass slide coated with patterned tape was immersed into glass etching solution at a certain speed to fabricate the microchannel with continuously decreasing height (from 60 to 5 μm). Third, two inlets and one outlet (0.5 mm in diameter) were drilled on the glass slide and the up layer of the CTC-ΔChip was finished. Finally, the two pieces of glass slides were bonded together after a dynamic heating and annealing process in a programmable muffle furnace.
