# Supplementary material for: Wedge-shaped microfluidic chip for circulating tumor cells isolation and its clinical significance in gastric cancer
Source: J Transl Med. 2018 May 23;16:139. doi: 10.1186/s12967-018-1521-8 (PMC5966930; doi:10.1186/s12967-018-1521-8)
Supplement: Supplementary file 2 — Additional file 2: Figure S1. Detailed structure of the microfluidic device. [file 12967_2018_1521_MOESM2_ESM.docx]

**Additional file 2**


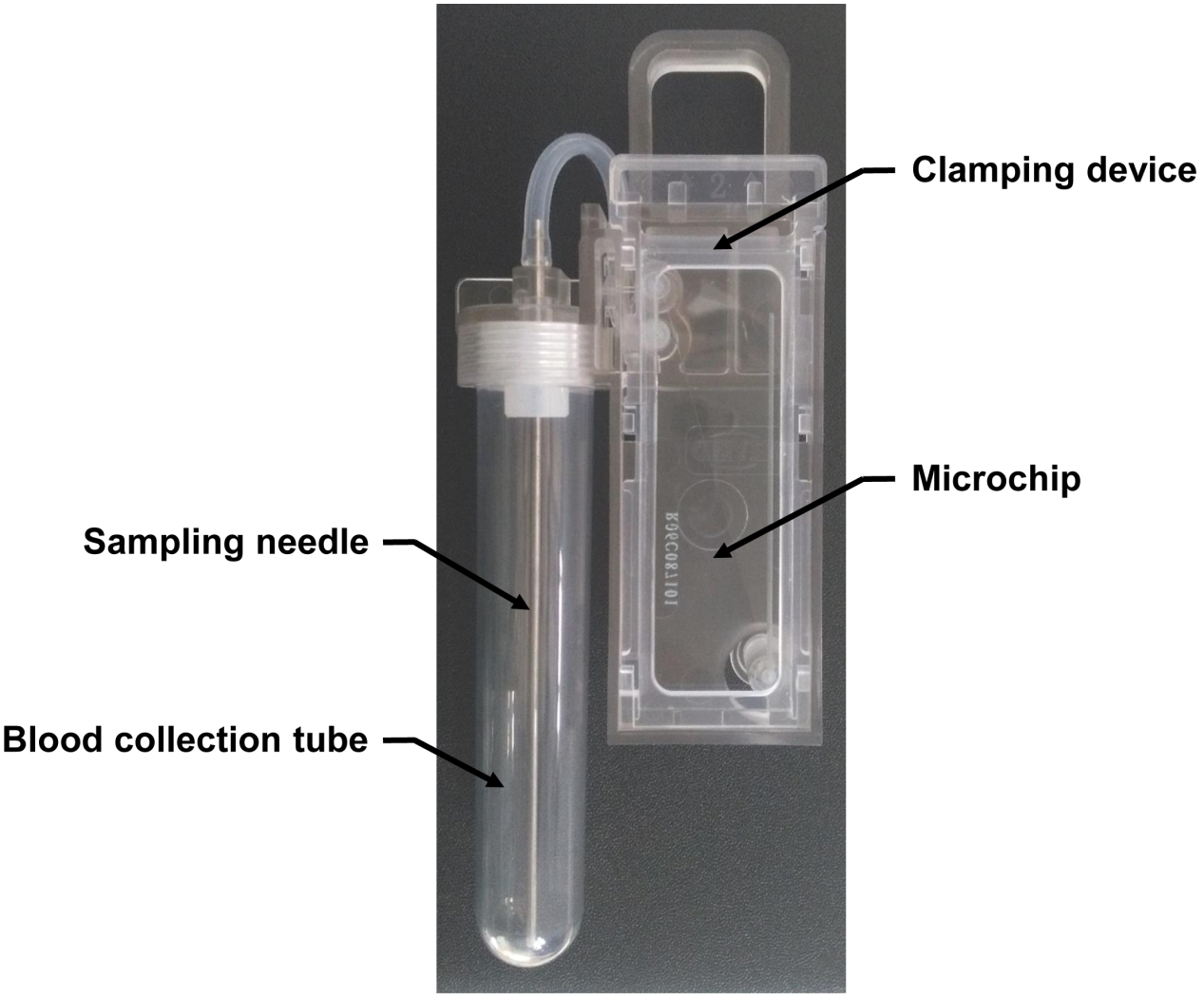


**Figure S1** Detailed structure of the microfluidic device. The microfluidic device was composed of four parts, including a blood collection tube, sampling needle, clamping device and CTC-ΔChip. As the core functional part of the microfluidic device, CTC-ΔChip was fixed by the clamping device. The sampling needle was responsible for drawing the sample of the blood collection tube into CTC-ΔChip to process, with the flow rate of 200 μL/min.
