# Supplementary material for: Wedge-shaped microfluidic chip for circulating tumor cells isolation and its clinical significance in gastric cancer
Source: J Transl Med. 2018 May 23;16:139. doi: 10.1186/s12967-018-1521-8 (PMC5966930; doi:10.1186/s12967-018-1521-8)
Supplement: Supplementary file 4 — Additional file 4: Table S1. Detailed clinical information and detected CTCs counts of 76 cancer patients. [file 12967_2018_1521_MOESM4_ESM.docx]

| **Table S1 Detailed clinical information and detected CTCs counts of 76 cancer patients.** | | | | | | | | | | | | |
| --- | --- | --- | --- | --- | --- | --- | --- | --- | --- | --- | --- | --- |
| **Cancer species** | **Patients**  **(n)** | | **Age (year)** | |  | **Sex** | |  | **TNM stage^a^** | | | |
|  |  |  | **Mean** | **Range** |  | **Male** | **Female** |  | **Ⅰ** | **Ⅱ** | **Ⅲ** | **Ⅳ** |
| Breast cancer | 20 | 53.0 | | 39-69 |  | 0 | 20 |  | 5 | 7 | 6 | 2 |
| Lung cancer | 15 | 55.5 | | 41-70 |  | 10 | 5 |  | 2 | 5 | 5 | 3 |
| Esophageal cancer | 13 | 55.0 | | 46-67 |  | 9 | 4 |  | 1 | 4 | 6 | 2 |
| Gastric cancer | 16 | 60.6 | | 45-71 |  | 10 | 6 |  | 3 | 4 | 6 | 3 |
| Colorectal cancer | 12 | 56.1 | | 32-68 |  | 9 | 3 |  | 2 | 3 | 5 | 2 |
| **^a^ 7th edition of AJCC/UICC classification system.** **CTCs, circulating tumor cells.** | | | | | | | | | | | | |

Additional file 4
