# Supplementary material for: Wedge-shaped microfluidic chip for circulating tumor cells isolation and its clinical significance in gastric cancer
Source: J Transl Med. 2018 May 23;16:139. doi: 10.1186/s12967-018-1521-8 (PMC5966930; doi:10.1186/s12967-018-1521-8)
Supplement: Supplementary file 5 — Additional file 5: Figure S2. CTCs images of various type of cancers. [file 12967_2018_1521_MOESM5_ESM.docx]

**Additional file 5**


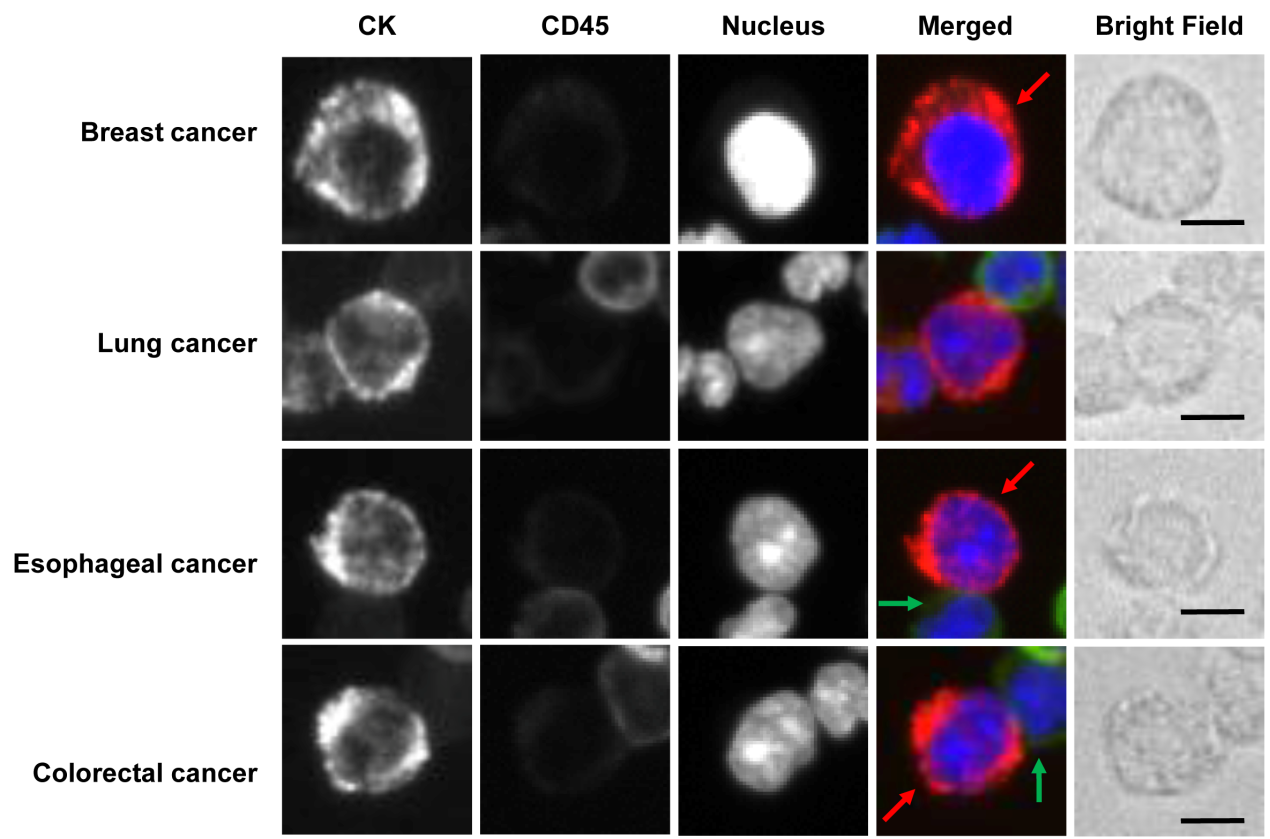


**Figure S2** CTCs images of various type of cancers. Staining with three-color immunocytochemistry method based on CK, CD45, and nuclear staining for isolated CTCs from cancer patients. Scale bars, 10 μm.
