# Supplementary material for: Wedge-shaped microfluidic chip for circulating tumor cells isolation and its clinical significance in gastric cancer
Source: J Transl Med. 2018 May 23;16:139. doi: 10.1186/s12967-018-1521-8 (PMC5966930; doi:10.1186/s12967-018-1521-8)
Supplement: Supplementary file 6 — Additional file 6: Figure S3. Number of captured CTCs from blood samples of various type of cancer patients. [file 12967_2018_1521_MOESM6_ESM.docx]

**Additional file 6**


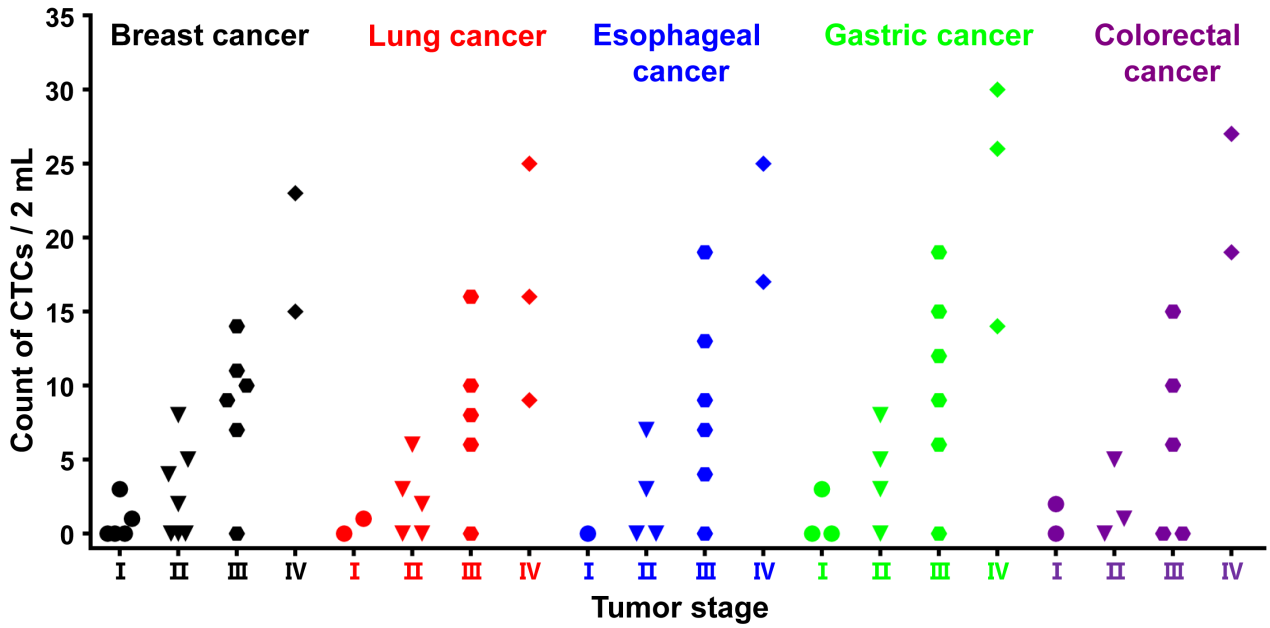


**Figure S3** Number of captured CTCs from blood samples of various type of cancer patients, including 20 breast cancers, 15 lung cancers, 13 esophageal cancers, 16 gastric cancers and 12 colorectal cancers.
