# Supplementary material for: Wedge-shaped microfluidic chip for circulating tumor cells isolation and its clinical significance in gastric cancer
Source: J Transl Med. 2018 May 23;16:139. doi: 10.1186/s12967-018-1521-8 (PMC5966930; doi:10.1186/s12967-018-1521-8)
Supplement: Supplementary file 7 — Additional file 7: Table S2. Association between CTCs and tumor markers. [file 12967_2018_1521_MOESM7_ESM.docx]

**Additional file 7**

| **Table S2 Association between CTCs and tumor markers** | | | | |
| --- | --- | --- | --- | --- |
| **Parameter** | **CTC + (n =30)** | **CTC- (n = 10)** |  | **P value^b^** |
| CEA (ng/ml） | 39.93±5.10^a^ | 11.83±5.16 |  | 0.002 |
| CA153 (U/ml) | 19.84±1.44 | 16.88±1.97 |  | 0.118 |
| CA199 (U/ml) | 43.13±6.08 | 44.18±7.46 |  | 0.719 |
| CA724 (U/ml) | 6.63±0.93 | 4.04±0.52 |  | 0.241 |

**^a^ Mean±SEM, ^b^ P values from the Mann–Whitney test. CTCs, circulating tumor cells.**
